# The Treatment of Intussusception

**Published:** 1882-09

**Authors:** 


### THE TREATMENT OF INTUSSUSCEPTION.

In the September number of the *New York Medical Journal and Obstetrical Review*, Dr. W. R. Gillette, Physician to Bellevue Hospital, relates a case of intussusception in a child nine months old, relieved by injections of water, the admistration of chloroform by inhalation, and manipulation of the tumor felt through the abdominal wall. This, he states, is the third case of intussusception in infants which he has seen, and which he has been able to reduce by these means. He thinks that these cases, from the philosophy of their condition, and the necessary measures for relief, are best managed in the way indicated. In two other instances, in which he saw and advised this treatment, reduction was utterly impossible under the other methods tried. The children in each of these cases were held while struggling, and the injections forced into them against all voluntary and involuntary efforts which they could make. He deems the admistration of chloroform almost absolutely necessary in these cases. The reason is not difficult to find, inasmuch as, while it gives us such perfect control of the patient, it also eliminates the element of muscular spasm. Moreover, massage is a powerful adjuvant to the hydrostatic pressure of water in these cases. In the first two cases the obstruction was not overcome until massage also was employed.

